# Molecular Docking and Simulation-Binding Analysis of Plant Phytochemicals with the Hepatocellular Carcinoma Targets Epidermal Growth Factor Receptor and Caspase-9

**DOI:** 10.3390/molecules28083583

**Published:** 2023-04-20

**Authors:** Ghulam Mustafa, Shumaila Younas, Hafiza Salaha Mahrosh, Mohammed Fahad Albeshr, Eijaz Ahmed Bhat

**Affiliations:** 1 Department of Biochemistry, Government College University Faisalabad, Faisalabad 38000, Pakistan; 2 Department of Biochemistry, University of Agriculture Faisalabad, Faisalabad 38000, Pakistan; 3 Department of Zoology, College of Science, King Saud University, P.O. Box 2455, Riyadh 11451, Saudi Arabia; 4 Centre de Biologie Structurale (CBS), INSERM, CNRS, Université de Montpellier, 34090 Montpellier, France

**Keywords:** liquoric acid, phytochemicals, HIF1a, Lipinski’s role of five, ADMET

## Abstract

Among primary liver cancers, hepatocellular carcinoma (HCC) is one of the most common forms and it has been categorized as the joint-fourth largest reason of cancer-related deaths globally. Different factors such as alcohol abuse, hepatitis B and C, viral infections, and fatty liver diseases are mainly related to the pathogenesis of HCC. In the current study, 1000 total various plant phytochemicals were docked to proteins involved in HCC. The compounds were docked to the active site amino acids of epidermal growth factor receptor and caspase-9 as receptor proteins in order to explore their inhibiting potential. The top five compounds against each receptor protein were explored as potential drug candidates on the basis of their binding affinity and root-mean square deviation values. The top two compounds against each protein were found to be liquoric acid (S-score −9.8 kcal/mol) and madecassic acid (S-score −9.3 kcal/mol) against EGFR, and limonin (S-score −10.5 kcal/mol) and obamegine (S-score −9.3 kcal/mol) against the caspase-9 protein. The selected phytochemicals were further assessed through drug scanning using Lipinski’s rule of five to explore their molecular properties and druggability. According to the ADMET analysis, the selected phytochemicals were found to be non-toxic and non-carcinogenic. Finally, the molecular dynamics simulation study revealed that liquoric acid and limonin were stabilized within the binding pockets of EGFR and capase-9, respectively, and stayed firmly bound throughout the simulation. In light of the current findings, the phytochemicals reported in this study, especially liquoric acid and limonin, could be used as potential drugs for the treatment of HCC in the future.

## 1. Introduction

Hepatocellular carcinoma (HCC) is characterized as the fourth most fatal malignancy and the leading death-related cancer globally [1]. The progression of HCC is greatly associated with a variety of factors and infections, including hepatitis B and C virus. It is still difficult to diagnose cancer at an early stage due to the lack of diagnostic methods. Treating the tumor becomes challenging when the cancer enters into the metastasis stage [2]. Alcohol abuse is a high risk factor that is directly associated with HCC onset and progression compared to any infection [3]. Despite approaches such as liver transplantation, radiotherapy, chemotherapy, screening, and many alternative combined therapies, the eradication of the tumor is still challenging and the mortality rate is continuously increasing [4].

The health of the human liver is vital to preserve the usual physiological systems including metabolism of proteins, fat, and carbohydrates, blood filtration, immunological functions, storage of vitamins and minerals, bile formation, and angiotensinogen synthesis for maintaining blood pressure [5]. To increase the life span of patients, the initial recognition of liver cancer and better combinations, which are liable to stop the development of HCC, are important to recognize and decrease instances of HCC-linked infection(s) [6]. Liver cirrhosis is not only linked to liver infection but it is also reported in non-alcoholic fatty liver disease (NAFLD) and non-alcoholic steatohepatitis (NASH) cases. This predicts the interrelation of HCC with NAFLD [7]. HCC and ICC (i.e., intrahepatic cholangiocarcinoma) are two histological forms of liver cancer with 75% of HCC and 12–15% of ICC clinical cases reported over the globe [8]. HCC is the cause of 1% of the liver malignances, while secondary liver metastasis or liver cancer causes the rest of the instances of liver cancer. The progression of HCC in hepatocytes is mainly due to inflammation, oxidative stress, and primary liver disease, while ICC progresses in cholangiocytes, which are populated with epithelial cells in the intrahepatic bile duct as a bile tree [9].

Plants contribute towards the natural source of a variety of biologically active constituents, which can be used as the leading drug candidates for HCC after careful analyses. Plant-derived compounds have proven anticancer potentials for the treatment of a variety of malignancies [10]. Studies have emphasized the introduction and usage of numerous plant-derived compounds as chemotherapeutic drugs [1].

The molecular docking approach characterizes the atomic level interactions between plant phytochemicals as ligand molecules and different proteins as receptor or target molecules to explore the binding potential of the ligands to target binding sites [11]. Despite the recent advancements, such as screening methods, new technologies, and therapies, HCC remains the deadliest cancer with a low survival rate. The ligand molecules recognized through molecular docking are more probable to progress to the next level in the process of drug development. The late diagnosis due to complex gene network and side effects of drugs are the most common reasons of treatment failure. A chief concern must therefore be to provide effective and competent care to patients with end-stage liver disease and to progress the novel anti-HCC medications. Therefore, there is a dire need for drugs that are less toxic and naturally available. The objective of the current study was therefore to target the receptor proteins that are involved in the pathogenesis of HCC. Different targets such as transcription factors, apoptotic proteins, growth factor receptors (GFRs), cell division protein kinase, mitogen-activated protein kinases, and serine/threonine protein kinases could be used as receptor proteins. In the current study, EGFR and caspase-9 were used as the target proteins. The information from this current investigation is valuable in terms of exploring novel compounds that could be used in developing drugs against HCC in the future.

## 2. Results

The three-dimensional (3D) structures of epidermal growth factor receptor (EGFR) and caspase-9 were retrieved from the PDB database as receptor or target proteins. A total of 1000 phytochemicals were used as ligands and explored for their binding interactions with the amino acids of the active sites of the selected proteins involved in hepatocellular carcinoma through molecular docking studies.

### 2.1. Interaction Analysis

A PyRx virtual screening tool and BIOVIA Discovery Studio were used for the docking and visualization of 2D patterns of ligand–target protein interactions, respectively. The top drug candidates were predicted based on their binding affinities and RMSD values. PyRx displayed the occupancy of the binding pocket of the target molecule by the ligand via the conformation scores. Among the one thousand phytochemicals, the top five compounds were selected separately against each receptor protein on the basis of the minimum binding score and RMSD values (Table 1).

**Figure 1:**
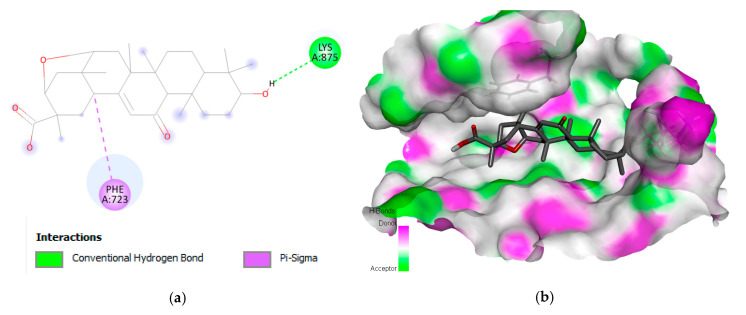
Interaction (**a**) and binding pattern (**b**) of liquoric acid with EGFR as a receptor.

Liquoric acid revealed interactions with PheA:723 and LysA:8775 amino acids of EGFR protein with a binding or S-score of −9.8 kcal/mol (Figure 1). A conventional H-bond with LysA:875 was found in the complex and a Pi–sigma (PheA:723) was also observed in the complex. The Pi–sigma interactions (i.e., Pi–alkyl and Pi–sulfur) help to intercalate the drug in the binding pocket of the receptor as they are largely involved in the charge transfer.

**Figure 2:**
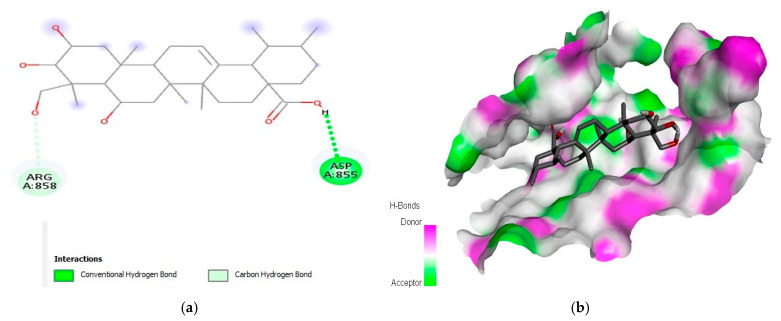
Interaction (**a**) and binding pattern (**b**) of madecassic acid with EGFR as a receptor.

Similarly, the second best phytochemical madecassic acid (S-score of −9.3 kcal/mol) revealed interactions with AspA:855 and ArgA:858 amino acids at the binding site of EGFR (Figure 2). Other phytochemicals such as berbamine, obamegine, and isotetrandrine also interacted with PheA:723 as a common interacting residue of the binding pocket of the EGFR protein. The amino acids AspA:837, AsnA:842, AspA:855, ArgA:858, and ProA:877 were found to be common in different complexes (Figures S1–S3).

**Figure 3:**
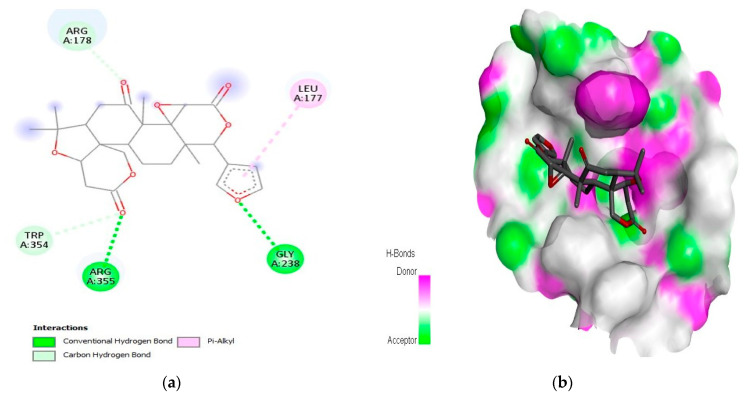
Interaction (**a**) and pattern of binding (**b**) of limonin with caspase-9 as a receptor.

**Figure 4:**
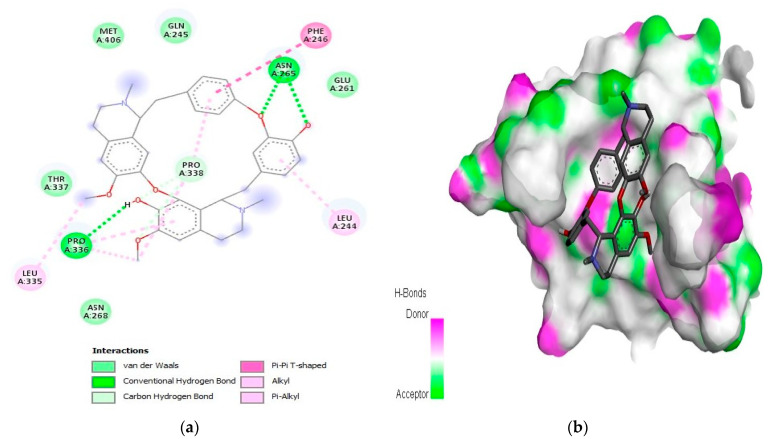
Interaction (**a**) and pattern of binding (**b**) of obamegine with caspase-9 as a receptor.

For the caspase-9 target protein, the best phytochemical limonin with a binding affinity of −10.5 kcal/mol interacted with LeuA:177, ArgA:178, GlyA:238, TrpA:354, and ArgA:355 amino acids of the active pocket (Figure 3). Two conventional H-bonds (i.e., GlyA:238 and ArgA:355) were observed in this complex. Similarly, the second best phytochemical obamegine showed interactions with LeuA:244, PheA:246, AsnA:265, LeuA:335, ProA:336, and ProA:338 residues of the active site (Figure 4). Two conventional H-bonds (i.e., AsnA:265 and ProA:336) were also observed in this complex. The remaining phytochemicals, i.e., liquoric acid, 3-O-caffeoyloleanolic acid, and botulin, also showed strong binding interactions to the amino acids of the caspase-9 active site (Figures S4–S6). Among the best-selected phytochemicals, the compounds liquoric acid, limonin, berbamine, isotetrandrine, and obamegine showed strong interactions with both receptor proteins (i.e., EGFR and caspase-9).

### 2.2. Druggability Analyses

The selected drug candidates must follow at least four rules of Ro5 to be potential drug candidates. The probability of oral bioavailability of these compounds will be high and could be considered for further experimental studies, e.g., in animal models such as rats [12]. Among all the top compounds, only limonin followed all the drug-like parameters according to Ro5. The compound liquoric acid violated only one rule, while the other compounds (i.e., madecassic acid, obamegine, botulin, lupeol, and berbamine) violated more than one rule of the Ro5 (Table 2). If a compound violates no rules or one out of the five, it could be considered as a potential drug candidate [13]. The admetSAR tool was also employed for further assessing the pharmacological potential of the best-selected phytochemicals through ADMET-based attributes from a medical perspective. The selected compounds fulfilled most of the criteria and confirmed their bioavailability (Table 3). All selected phytochemicals were found to be non-carcinogenic, but only three phytochemicals (i.e., berbamine, isotetrandrine, and obamegine) were found to be Ames toxic. Based on the overall drug profiling, the preferred or selected phytochemicals reported in this study accomplished the criteria of being potential drug candidates.

### 2.3. MD Simulation

**Figure 5:**
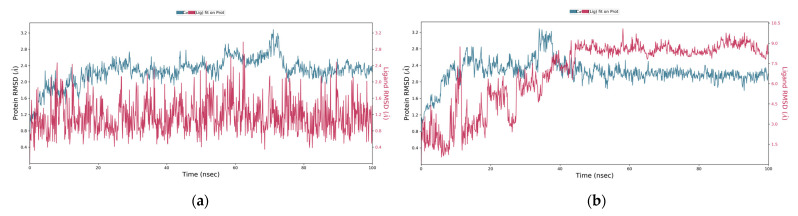
RMSD values of liquoric acid and limonin with receptor proteins. (**a**) RMSD of the C-alpha atoms of EGFR and liquoric acid; (**b**) RMSD of the Cα atoms of caspase-9 and limonin with time. The variation in RMSD of receptor protein is shown on the *y* axis (on left) through time. The variation in RMSD of the ligand is shown on the *y* axis (on right) through time.

To check the overall stability and/or flexibility of the ligand-protein complexes, molecular dynamics simulations of 100 ns were run using Desmond (Schrödinger LLC, New York, NY, USA). Two phytochemicals (i.e., liquoric acid and limonin) were selected for MD simulation studies because they showed strong binding interactions with EGFR and caspase-9 receptor proteins, respectively, and were proven as potential drug candidates. MD simulations were studied for docking complexes of liquoric acid-EGFR and limonin-caspase-9 in replicates. From the molecular dynamics trajectories, RMSD (root-mean-square deviation) was used to determine the fluctuations of a ligand within the active site of the receptor protein. For Cα atoms of the protein–ligand complex, the evolution of the RMSD values over time is presented in Figure 5. The plot of RMSD values shows that the liquoric acid-EGFR complex was stable throughout the simulation time of 100 ns and the ligand remained firmly bound to the receptor (Figure 5a). In its replicate, the plot of RMSD values shows that the liquoric acid-EGFR complex got stable at 15 ns (Figure S7a). The RMSD of the receptor was increased at 40 ns, but later, the fluctuations in the RMSD values remained within 0.6 Å for the length of the MD simulation and this is absolutely acceptable [14]. Furthermore, the fluctuation of the liquoric acid is not higher than the EGFR, so it could be concluded that liquoric acid is stabilized within the binding pocket of the EGFR. The findings show that the ligand remained strongly connected to the EGFR protein during the entire period of the MD simulation.

Similarly, the plot of RMSD values of limonin-caspase-9 complex showed that the complex reached stability at 43 ns (Figure 5b). Fluctuations were observed until 43 ns but for the remaining MD simulation time, the ligand was firmly bound to the receptor protein. In the replicate, the RMSD plot showed a similar trend (Figure S7b). It showed that the ligand shifted its mode after 40 ns and then stabilized for the remaining MD simulation time. Fluctuations were observed at 80 to 85 ns, but these were not dramatic ones and the ligand was firmly bound to the caspase-9 protein.

**Figure 6:**
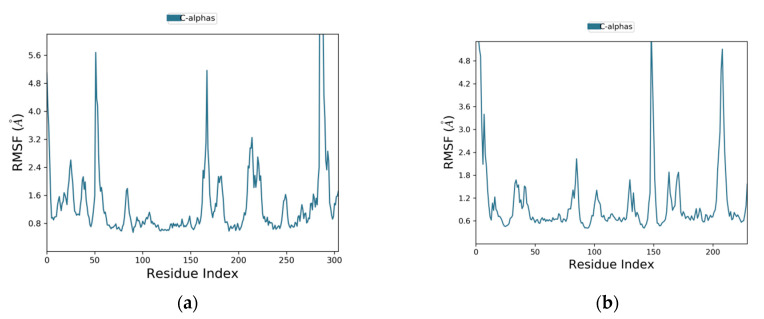
The plots of residue wise root mean square fluctuation (RMSF). (**a**) RMSF plot of EGFR and (**b**) RMSF plot of caspase-9 as receptor proteins.

The root mean square fluctuation (RMSF) plots are given in Figure 6. The fluctuations of the amino acids of EGFR were between 0.8 and 5.7 Å (Figure 6a), while the fluctuations of the amino acids of caspase-9 were between 0.6 and 4.9 Å (Figure 6b). In RMSF plots, the peaks are showing the portions of the receptor proteins that exhibited maximum fluctuations during the MD simulation. Generally, the N and C terminals, which are known as protein tails, show higher fluctuations than any other rigid structures, such as the α-helices and β-strands. Usually, the α-helices and β-strands are stiffer compared to nonstructured sections in a protein and therefore they show less fluctuations.

**Figure 7:**
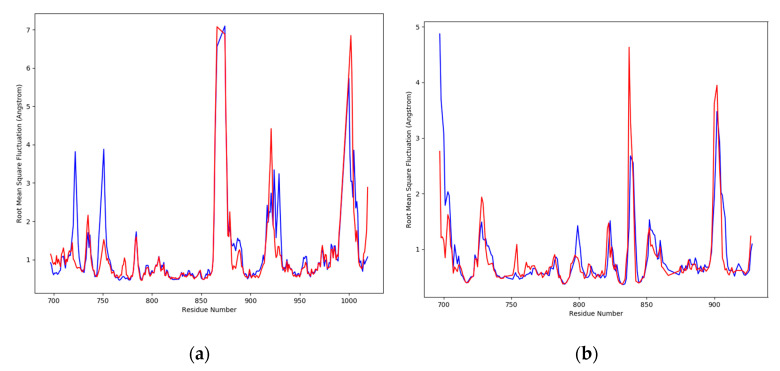
The plots of residue wise root mean square fluctuation (RMSF) of receptor proteins in apo and bound states. (**a**) RMSF plot of EGFR protein (in apo and bound state) and (**b**) RMSF plot of caspase-9 protein (in apo and bound state). The red peaks are showing apo proteins, while the blue peaks are showing values for bound states.

To reveal residue-wise fluctuations between the ligands complexed with receptor proteins and apo proteins, the RMSF values were plotted. A dynamic movement was clearly shown by the RMSD deviations for the complex and apo proteins, which were found in the loop regions. To reveal the amino acids that were involved in the fluctuations, the RMSF plot is shown in Figure 7. Each position of the amino acids was determined by their deviation values for the simulation time of 100 ns. At the binding area of EGFR-liquoric acid, several residues showed different flexibility values between the bound and apo state. For the EGFR apo protein, the amino acids in the regions 855–880th, 912–925th, and 990–1005th showed deviations of up to ~7 Å, while the amino acids of other regions were found to be deviating from ~1–4 Å (Figure 7a). In the comparison of apo RMSF values with the complex protein RMSF, the ligand liquoric acid also shows deviations in the 855–880th, 912–930th, and 990–1005th positions from ~1–7 Å. Other than these regions, the amino acid positions, 712–725th and 745–755th, show deviations from ~1–3.8 Å (Figure 7a), which shows the rigidity of the protein in the complex state.

Similarly, for the caspase-9 apo protein, the amino acids in the 830–845th and 855–910th positions were found to be the most flexible regions, which showed deviations up to ~4.5 Å and the amino acids of other regions deviated from ~1–2 Å (Figure 7b). When the apo RMSF values were compared to the complex protein RMSF, the ligand limonin also showed deviations in these regions from ~1–3.5 Å, while the amino acids of other regions showed deviations from ~1–1.5 Å (Figure 7b), which showed that the protein was rigid in the bound state.

The amino acids that belong to the regions of N- and C-terminals and/or loop areas in the EGFR (Figure S8) and caspase-9 (Figure S9) receptor proteins exhibited higher peaks in their MD trajectories. The interaction or binding of the ligand to the target protein will be stable if the RMSF values of the amino acids at the binding site are low. The secondary structure elements (i.e., α-helices and β-strands) are monitored during MD simulation. In the graphs above in Figures S8 and S9, the distribution of the SSE (secondary structure elements) is shown by the residue index in the structure of the receptor protein. For each trajectory frame, the SSE composition and assignment of each residue are shown in the left and right graphs, respectively, throughout the simulation time. The interactions between protein–ligand complexes could be detected throughout the MD simulation.

**Figure 8:**
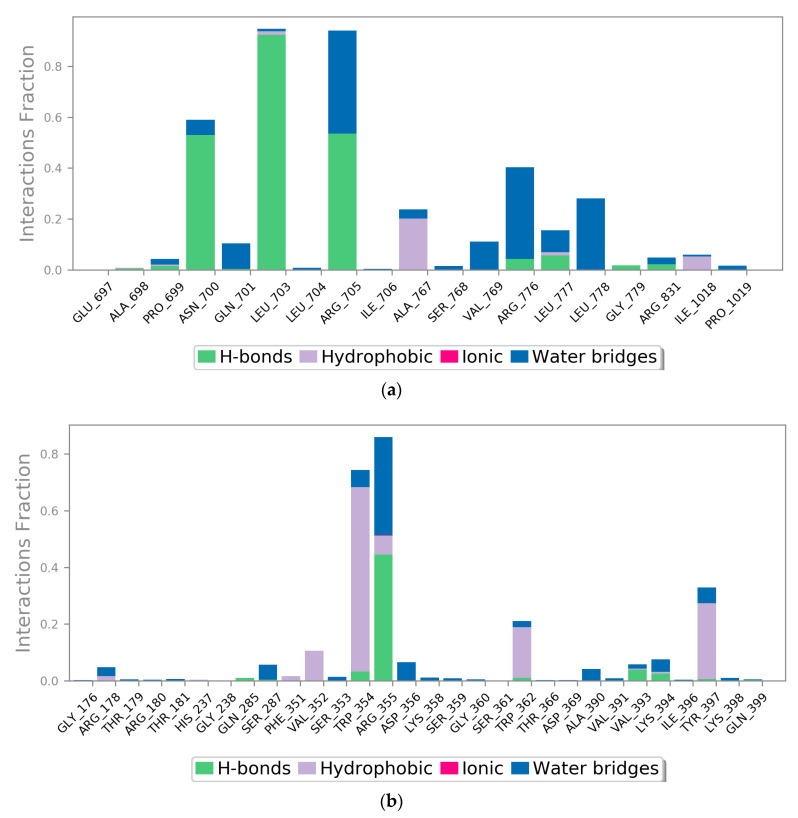
Protein–ligand contact histograms (hydrogen bonding, hydrophobic interactions, ionic interactions, and water bridges) of (**a**) EGFR-liquoric acid complex and (**b**) caspase-9-limonin complex.

There are four different types of protein–ligand interactions or contacts, which include H-bonds, water bridges, ionic interactions, and hydrophobic interactions. In Maestro, the Simulation Interaction Diagram panel was employed for the study of subtypes of each interaction type. Over the entire trajectory, the stacked bar charts were standardized, e.g., the value of 0.7 means that an interaction was retained for 70% of the MD simulation. Some amino acid residues in the target protein reveal interactions of same subtype with the ligands and therefore, values ≥ 1.0 are feasible. The MD simulation revealed that the majority of the significant interactions in the EGFR-liquoric acid complex were H-bonds and hydrophobic interactions (Figure 8a). For the EGFR-liquoric acid complex, Asn_700, Leu_703, and Arg_705 are the most important amino acids in terms of hydrogen bonding, while in terms of hydrophobic interactions, the amino acids Ala_767 and Ile_1018 are the most important residues. For the caspase-9-limonin complex, the majority of the significant interactions were hydrophobic and a few were H-bonds. Trp_354, Trp_362, and Tyr_397 are the most important residues with respect to hydrophobic interactions and Arg_355 is the most important residue with respect to hydrogen bonding (Figure 8b).

**Figure 9:**
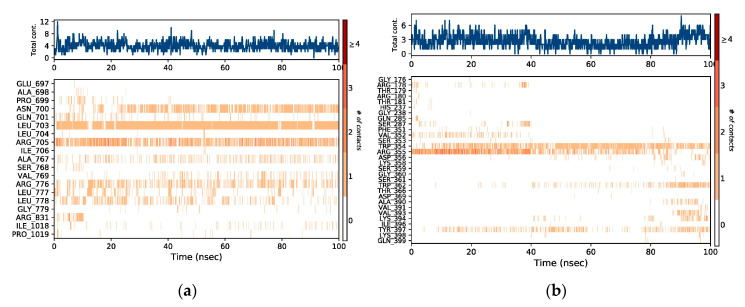
The representation is showing the timeline of the interactions and contacts (hydrogen bonding, hydrophobic interaction, ionic interaction, and water bridges) between (**a**) EGFR-liquoric acid complex and (**b**) caspase-9-limonin complex.

All types of interactions and contacts are exhibited through a timeline, as described above. In Figure 9, the panel above shows the total number of specific connections that are made by proteins with their respective ligands for the duration of the MD simulation. The lower panels of each trajectory frame show the residues of proteins that are interacting with their respective ligands. Some amino acids have made multiple connections to the ligand molecule, which are shown according to the scale by a deeper orange color shade at the right side of the graph.

**Figure 10:**
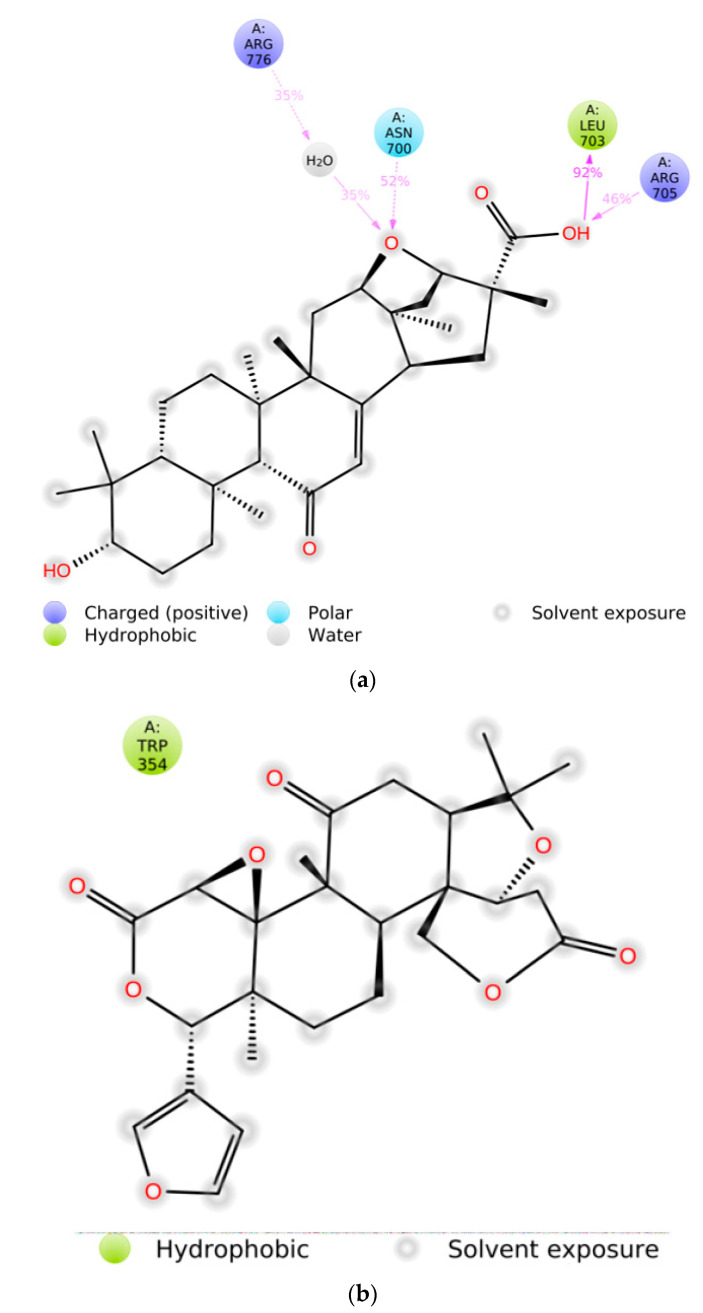
Interactions of ligand atoms to the amino acids of the receptor proteins. (**a**) Interaction of liquoric acid atoms with EGFR protein residues; (**b**) interaction of limonin atoms with caspase-9 protein residues.

The stacked bar charts were standardized for the trajectory course, which shows that if the value of contact is 1.0, then it means that the exact interaction is retained for 100% of MD simulation period. Values > 1.0 are also possible, as some of the amino acids interact multiple times to the ligand molecule through the same subtype of interactions. The interactions of liquoric acid with EGFR are shown in Figure 10a and the interactions between limonin and caspase-9 are shown in Figure 10b. The interactions that lasted for longer than 30% of the MD simulation duration in their respective trajectories (i.e., 0.00 to 100 ns) are shown in the figure.

**Figure 11:**
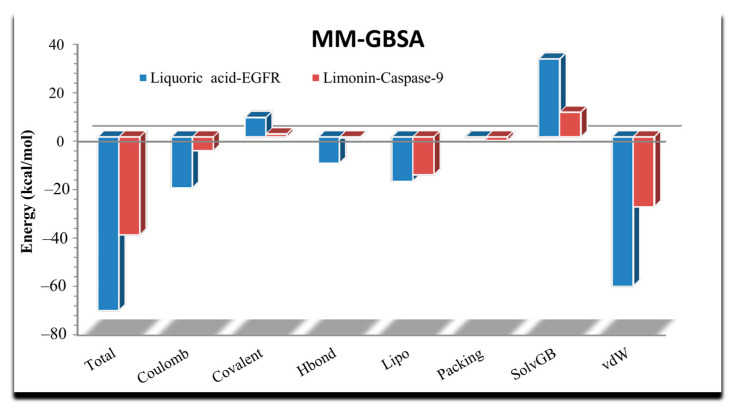
MM-GBSA analysis to predict binding energy between receptors and the ligands (liquoric acid and limonin). The total binding free energy of MM-GBSA was determined by adding seven energies given at the bottom of the chart.

The MM-GBSA (Molecular Mechanics Generalized Born Surface Area) was also determined for molecular dynamics simulation (Figure 11). For the liquoric acid-EGFR complex, the average ΔG value was found to be −71.67, the ΔG standard deviation was 8.02, and the ΔG range was found to be −63.75 to −89.45 (Table S1). Similarly, the average ΔG for the limonin-caspase-9 complex was found to be −40.44. The ΔG standard deviation was 7.79, and the ΔG range was found to be −38.48 to −39.38 (Table S2). The MM-GBSA analysis revealed that most of the residues of both receptor proteins near their binding sites to the ligands showed a significant contribution toward the stabilization of ligands, which is depicted by lower values of their binding energies.

## 3. Discussion

The human liver is comprised of 2.5% of average body mass and therefore is the largest organ in the body. It participates in multiple vital bodily functions such as the metabolism of proteins, lipids, carbohydrates, and the detoxification of compounds [15]. Different toxins can induce liver injury or chronic liver infections (e.g., hepatitis B and C), which ultimately transform into liver malignancies [16]. Recently, the FDA has approved the use of the combined drugs sorafenib, atezolizumab, and bevacizumab to treat hepatocellular carcinoma (HCC) [17]. A synthetic compound sorafenib targets VEGFR-2/PDGFR-beta signaling cascade and growth signaling, and therefore blocks tumor angiogenesis. Sorafenib blocks RAF kinase enzyme, which is an important part of the RAF/MEK/ERK signaling pathway that is involved in the regulation of cell division and proliferation [18].

To prevent and cure human HCC still remains a global issue because of the inadequate diagnostic biomarkers and therapeutic strategies. Different therapeutic strategies have been used, such as chemotherapy and radiotherapy, which are still unable for providing substantial improvements for the survival of patients with liver cancer. Since the last few decades, herbal products derived from various plant sources have been playing important roles in terms of preventing different cancers and developing therapeutic agents against these cancers [19].

Computational biology helps to predict the binding possess of different potential ligands within the active sites of target proteins as pockets. Virtual screening and molecular docking are often employed to discover new medicines [20]. Molecular docking methods aim to identify the correct position of a ligand molecule within the binding or active site of the target or receptor protein to investigate the right position of the ligand molecule with the receptor protein. The methods are also used for the prediction of the binding patterns of the ligand with the receptor protein [21].

Although, the exact molecular mechanisms that lead to the occurrence, progression, and metastasis of HCC are not yet fully understood. However, several genes, factors, and pathways are involved in the development of HCC. The EGFR is a member of the ErbB family of receptors and is a transmembrane tyrosine kinase receptor. The ErbB family is consisted of four members, namely, EGFR (ErbB1), HER2/neu (ErbB2), HER3 (ErbB3), and HER4 (ErbB4). EGFR has a distinctive structure which is consisted of three different domains (i.e., a transmembrane domain, an extracellular ligand-binding domain, and a cytoplasmic domain that contains a region of tyrosine kinase with autophosphorylation sites). Previous research has suggested that EGFR is overexpressed in 60% of NSCLC cases and is correlated with a poorer prognosis [22]. Similarly, caspase-9 is a protease that initiates apoptosis through the intrinsic or mitochondrial pathway. It becomes activated at sites where multiple proteins are involved in the activation process [23]. The mitogen-activated protein (MAP) kinase superfamily includes extracellular signal-regulated kinases-1 and 2 (ERK1/2), which play a key role in regulating cell proliferation and survival. ERK1/2 proteins are known to prevent apoptosis, which is a form of programmed cell death, by deactivating proapoptotic proteins. In particular, ERK proteins have been observed to promote cell survival by blocking the activity of caspase-9 [24].

In the current study, a total of 1000 phytochemicals from various plants were selected to target the EGFR and caspase-9 proteins for the treatment of HCC. Among all the docked compounds, the top five compounds against each target protein were selected on the basis of their binding affinity through interactions to the amino acids present in the active sites of the selected target proteins. The best phytochemical in the case of EGFR was liquoric acid (docking score of −9.8 kcal/mol), which interacted with amino acids PheA:723 and LysA:875 of the EGFR protein. Likewise, madecassic acid (the second best phytochemical in the case of EGFR) interacted with AspA:855 and ArgA:858 amino acid residues of the active pocket of the EGFR with a binding affinity of −9.3 kcal/mol. Similarly, for caspase-9 protein, the phytochemical limonin with a binding score of −10.5 was found to be the top compound and it interacted with amino acids LeuA:177, ArgA:178, GlyA:238, TrpA:354, and ArgA:355 of the active site of the receptor protein caspase-9. Following limonin, obamegine with a binding score of −9.3 kcal/mol interacted with caspase-9 protein by binding to LeuA:244, PheA:246, AsnA:265, LeuA:335, ProA:336, and ProA:338 amino acids of its binding pocket.

Ghorab et al. [25] displayed the potential of a novel series of S-alkylated quinazolinone compounds as drugs against HCC. The results of their study showed that compounds **8** and **9** had the highest docking scores (−9.38 kcal/mol and −9.88 kcal/mol, respectively) and binding interactions among all the compounds. Compound **8** showed interaction with Met769 as a hydrogen bond acceptor via the carbonyl group of quinazoline and through a hydrophobic bond of Lys721 via a benzene ring attached to the amide. Compound **9** also showed an interaction with Met769 by the carbonyl group of amides, Thr766 as a hydrogen bond acceptor by nitrogen of quinazoline, and Lys721 through the carbonyl of quinazoline. In another study, Siddiqui et al. [26] represented the molecular docking of eight nominated phytochemicals viz. β-sitosterol, glucotropaeolin, O-ethyl-4-(α-L-rhamnosyloxy)-benzyl) carbamate, moringyne, moringin, niazimicin, pterygospermin, and niazirin of *Moringa oleifera* fruit, with apoptosis executioner caspase-3 protein. On the base of binding interactions and docking scores, benzyl glucosinolate (docking score of −8.4 kcal/mol) interacted with Tyr204, Trp206, Thr62, Arg207, Phe250, and Phe265 residues of caspase-3 protein binding pocket. Similarly, Suganya and Anuradha [27] docked astaxanthin and sorafenib to various apoptotic proteins which are involved in HCC. The astaxanthin interacted with residues Leu718, Phe723, Phe723, Glu804, and His805 of EGFR protein. Similarly, astaxanthin showed interactions with Phe348, Phe351, Ile396, Tyr397, Met400, Cys402, and Ile403 amino acid residues of caspase-9 protein.

Khalid et al. [28] represented a multitarget network pharmacology approach to explore the mechanisms of *S. surattense* to target HCC. A molecular docking analysis revealed the potential of quercetin to inhibit the target proteins involved in the pathogenesis of HCC. The result of their study indicated the pharmacological effects and potential molecular processes of *S. surattense* because of the interactions of its active ingredients with their therapeutic targets. They concluded that *S. surattense* active chemicals operate on possible genes as well as their influencing pathways to offer a network analysis in system pharmacology, which is crucial for developing new and novel drugs against HCC.

The drug-like analysis of selected compounds in this study displayed the potential of these compounds as anticancer drugs. Limonin followed all the drug-like parameters that are necessary to become a potential drug candidate to treat or suppress HCC. Liquoric acid violated only one rule and it was also a potential candidate in the suppression/treatment of HCC. The remaining phytochemicals violated two or three rules and could not be considered as good drug candidates. Similarly, the ADMET properties of a compound are also an important challenge in the route of drug development. The ADMET profiling of the best-selected compounds displayed different models including human intestinal absorption, P-glycoprotein substrate, renal organic cation transporter, and CaCO_2_ permeability that supported a strong ability of the selected compounds for their potential as drug candidates. Cytochrome P450 (CYP) is involved in in fatty acids, bile acids, and the metabolism of drug steroids. All the selected potential drug candidates were found to be non-toxic and non-carcinogenic, except for berbamine, isotetrandrine, and obamegine, which were predicted to be Ames toxic. Overall, the selected phytochemicals are good, potential drug candidates to treat HCC in the future.

## 4. Materials and Methods

The current study involves the docking of phytochemicals from different medicinal plants against EGFR and caspase-9 as target proteins to treat or suppress HCC. PyRx software was employed to perform molecular docking to access the binding patterns of ligand–receptor interactions.

### 4.1. Collection and Optimization of Bioactive Compounds

For the current study, a total of 1000 biologically important active phytochemicals were collected from the literature and different databases. The IMPPAT (Indian medicinal plants, phytochemistry and therapeutics) database [29] (https://cb.imsc.res.in/imppat/; accessed on 2 December 2022), and TCMSP (the traditional Chinese medicine systems pharmacology) database [30] (https://tcmsp-e.com/tcmsp.php; accessed on 2 December 2022) were used to search the active phytochemicals with reported anticancer activity. PubChem database [31] (https://pubchem.ncbi.nlm.nih.gov/; accessed on 4 December 2022) was used to retrieve the chemical structures of these plant phytochemicals in .sdf format. The phytochemicals’ energy was minimized before the molecular docking study.

### 4.2. Retrieval and Preparation of Receptor Proteins

The epidermal growth factor receptor (EGFR) and caspase-9 were selected as receptor proteins and used for molecular docking studies. The three-dimensional (3D) structures of receptor proteins (i.e., EGFR with PDB ID: 4LQM and caspase-9 with PDB ID: 2AR9) were downloaded from Protein Data Bank in .pdb format (https://www.rcsb.org/; accessed on 8 December 2022). The binding pockets of the receptor proteins were predicted using Molecular Operating Environment software [32]. To further prepare the receptor proteins for molecular docking, already bound ligand(s) were removed (if any), the water molecules were deleted, hydrogen atoms were added, and 3D protonation and energy minimization was performed to further prepare the receptor proteins for molecular docking.

### 4.3. Molecular Docking

The molecular docking study was performed to obtain compatible leading drug candidates for HCC treatment. PyRx software [33] was used for molecular docking of ligands to the active amino acids of the binding pocket of the receptor proteins. The Discovery Studio [34] software was used for the visualization of interactions between the receptor proteins and key active compounds. After docking, the conformations with best docking scores and RMSD (root-mean-square deviation) values were selected for further studies.

### 4.4. Drug Scanning through Pharmacokinetics Parameters

The assessment of druggability is an important step to reveal drug-like behavior of a compound as a potential drug candidate. The druggability assessment of the top phytochemicals was performed using SwissADME [35]. An online server admetSAR was employed to evaluate properties related to pharmacokinetics and pharmacodynamics. The ADMET (i.e., absorption, distribution, metabolism, excretion, and toxicity) profiling was checked. Parameters such as blood–brain barrier, carcinogenicity, human intestinal absorption, Ames toxicity, and CaCo-2 permeability help to predict the drug-like behavior of a drug candidate from the clinical biochemistry perspective. The Lipinski’s rule of five (Ro5) states that a drug candidate should have molecular mass <500 g/mol, hydrogen bond donors should be ≤5, hydrogen bond acceptors should be ≤10, logP should be ≤5, and the molecular refractivity index should be in the range of 40–130 [21]. The compounds were also tested for these parameters. The compounds could be processed further as leading drug candidates if they fully accomplished all these parameters.

### 4.5. Molecular Dynamics Simulation

The top compound against each receptor protein was further studied by molecular dynamic (MD) simulations. The Desmond (Schrödinger LLC) was employed for 100 nanoseconds to model MDs in replicates [36]. For the docking studies, the earliest phase of protein–ligand complex was used because the molecular docking study predicts the binding state of the ligand molecule in static situations. Molecular docking provides a static view of the best binding conformation of a molecule at the active site of a protein and is therefore important [37]. Typically, MD simulations integrate Newton’s classical equation of motion, computing the movement of atoms over time. The MD simulations were used in the current study for the prediction of ligand-binding status in the physiological environment [38,39].

To process the protein–ligand complex to optimize and minimize it, the Maestro or the Protein Preparation Wizard were employed. All systems were prepared using System Builder tool. A solvent model (i.e., transferable intermolecular interaction potential 3 points (TIP3P)) with an orthorhombic box was selected. The OPLS 2005 force field was used in the MD simulation study [40]. Counter ions were introduced to neutralize the models. To mimic the physiological conditions, NaCl (0.15 M) was added. The NPT ensemble with 1 atom pressure and 310 K temperature was selected for the simulation period. The models were relaxed before the MD simulation. After every 100 ps, the trajectories were saved to be examined. The MD simulation stability was verified via a comparison between the RMSD values of the protein and the ligand over time.

## 5. Conclusions

Hepatocellular carcinoma remains difficult to treat despite advanced treatment. In the current study, molecular docking, druggability analyses, and MD simulation studies were conducted on EGFR and caspase-9 proteins with plant phytochemicals. The phytochemicals limonin and liquoric acid demonstrated the best binding patterns among all the ligand molecules against the EGFR and caspase-9 receptor proteins, respectively. Drug-likeness and ADMET analysis of both these drug candidates displayed their drug-like behavior for the treatment of HCC. Both phytochemicals were found to be non-toxic and non-carcinogenic. Finally, the MD simulation study explored the fact that liquoric acid and limonin stayed firmly connected to the target proteins (i.e., EGFR and caspase-9, respectively) throughout the simulation period and, therefore, could be considered for further experimental studies to explore their anticancer activities to treat or suppress HCC in the future.

## Figures and Tables

**Table 1:** Binding scores of top 5 phytochemicals with EGFR and caspase-9 receptor proteins.

Sr. No.	Ligand	Receptor	Binging Affinity (kcal/mol)	Interacting Amino Acids
1	Liquoric acid	EGFR	−9.8	PheA:723 and LysA:875
2	Madecassic acid		−9.3	AspA:855 and ArgA:858
3	Berbamine		−8.7	PheA:723, ValA:726, LysA:745, AspA:837, ArgA:841, AsnA:842, LeuA:844, and ThrA:854
4	Obamegine		−8.4	PheA:723, HisA:835, AsnA:842, AspA:855, ArgA:858, GlyA:874, and ProA:877
5	Isotetrandrine		−8.2	PheA:723, AspA:837, GlyA:857, LysA:875, ValA:876, and ProA:877
6	Limonin	Caspase-9	−10.5	LeuA:177, ArgA:178, GlyA:238, TrpA:354, and ArgA:355
7	Obamegine		−9.3	LeuA:244, PheA:246, AsnA:265, LeuA:335, ProA:336, and ProA:338
8	Liquoric acid		−9.1	TyrA:153 and LysA:409
9	3-O-caffeoyloleanolic acid		−8.7	ArgA:146, GlyA:147, IleA:154, and LeuA:155
10	Betulin		−8.3	ArgA:408 andLysA:409

**Table 2:** Drug-like properties of the best-selected phytochemicals.

Ligands	Molecular Properties †						
	Molecular Mass (≤500 Dalton)	Hydrogen Bond Donor (≤5)	Hydrogen Bond Acceptor (≤10)	Number of Rotatable Bonds (≤10)	Log P (≤5)	Molar Refractivity (40–130)	Violations
Limonin	470.51	0	8	1	2.55	116.17	0
Liquoric acid	484.67	2	5	1	4.48	135.82	1
Madecassic acid	504.70	5	6	2	3.59	140.40	2
Obamegine	594.70	2	8	2	4.84	177.14	2
Betulin	442.72	2	2	2	6.36	136.30	2
Lupeol	426.72	1	1	1	7.26	135.14	2
Berbamine	608.72	1	8	3	5.15	181.60	3
Isotetrandrine	622.75	0	8	4	5.41	186.07	3

LogP is the logarithm of octanol/water partition coefficient. † SwissADME, an online tool, was used to calculate molecular properties.

**Table 3:** ADMET-related drug-like parameters of the best-selected phytochemicals.

	Phytochemicals							
	Liquoric Acid	Limonin	Madecassic Acid	Berbamine	Isotetrandrine	Obamegine	Betulin	Lupeol
Absorption								
BBB	No	No	Yes	No	Yes	Yes	Yes	No
HIA	Yes	Yes	Yes	Yes	Yes	Yes	Yes	Yes
Caco-2 Permeability	No	No	No	Yes	Yes	Yes	No	No
PGS	No	No	No	Yes	Yes	Yes	No	No
PGI	No	Yes	No	Yes	Yes	Yes	No	No
Metabolism								
CYP3A4 substrate	Yes	Yes	Yes	Yes	Yes	Yes	Yes	Yes
CYP2C9 substrate	No	No	No	Yes	Yes	Yes	No	No
CYP2D6 substrate	No	No	No	Yes	Yes	Yes	No	No
CYP3A4 inhibition	No	Yes	No	No	No	No	No	No
CYP2C9 inhibition	No	No	No	No	No	No	No	No
CYP2C19 inhibition	No	No	No	No	No	No	No	No
CYP2D6 inhibition	No	No	No	No	No	No	No	No
CYP1A2 inhibition	No	No	No	No	No	No	No	No
Toxicity								
AMES Toxicity	No	No	No	Yes	Yes	Yes	No	No
Carcinogens	No	No	No	No	No	No	No	No

Blood–brain barrier (BBB); human intestinal absorption (HIA); P-glycoprotein substrate (PGS); P-glycoprotein inhibitor (PGI).

## Data Availability

Not applicable.

## Supplementary Materials

The following supporting information can be downloaded at: https://www.mdpi.com/article/10.3390/molecules28083583/s1. Figure S1: Interactions (a) and binding patterns (b) of berbamine with EGFR as a receptor; Figure S2: Interactions (a) and binding patterns (b) of obamegine with EGFR as a receptor; Figure S3: Interactions (a) and binding patterns (b) of isotetrandrine with EGFR as a receptor; Figure S4: Interactions (a) and binding patterns (b) of liquoric acid with caspase-9 as a receptor; Figure S5: Interactions (a) and binding patterns (b) of 3-O-caffeoyloleanolic acid with caspase-9 as a receptor; Figure S6: Interactions (a) and binding patterns (b) of betulin with caspase-9 as a receptor; Figure S7: Root mean square deviation (RMSD) values of liquoric acid and limonin with receptor proteins (replicate). (a) RMSD of the C-alpha atoms of EGFR and liquoric acid; (b) RMSD of the C-alpha atoms of caspase-9 and limonin with time. The variation of protein RMSD is shown on left *y* axis through time. The variation of ligand RMSD is shown on right *y* axis through time; Figure S8: Protein secondary structure element (SSE) distribution by residue index throughout the protein structure of EGFR. Red indicates α-helices, and blue indicates β-strands; Figure S9: Protein secondary structure element (SSE) distribution by residue index throughout the protein structure of caspase-9. Red indicates α-helices, and blue indicates β-strands; Table S1: MM-GBSA-binding energy calculations of liquoric acid with EGFR after every ten nanoseconds from molecular dynamics simulation trajectories; Table S2: MM-GBSA-binding energy calculations of limonin with caspase-9 after every ten nanoseconds from molecular dynamics simulation trajectories.
