# Supplementary material for: Molecular Docking and Simulation-Binding Analysis of Plant Phytochemicals with the Hepatocellular Carcinoma Targets Epidermal Growth Factor Receptor and Caspase-9
Source: Molecules. 2023 Apr 20;28(8):3583. doi: 10.3390/molecules28083583 (PMC10143645; doi:10.3390/molecules28083583)
Supplement: Supplementary file 1 [file molecules-28-03583-s001.zip › molecules-2315135-supplementary.pdf]

Supplementary file

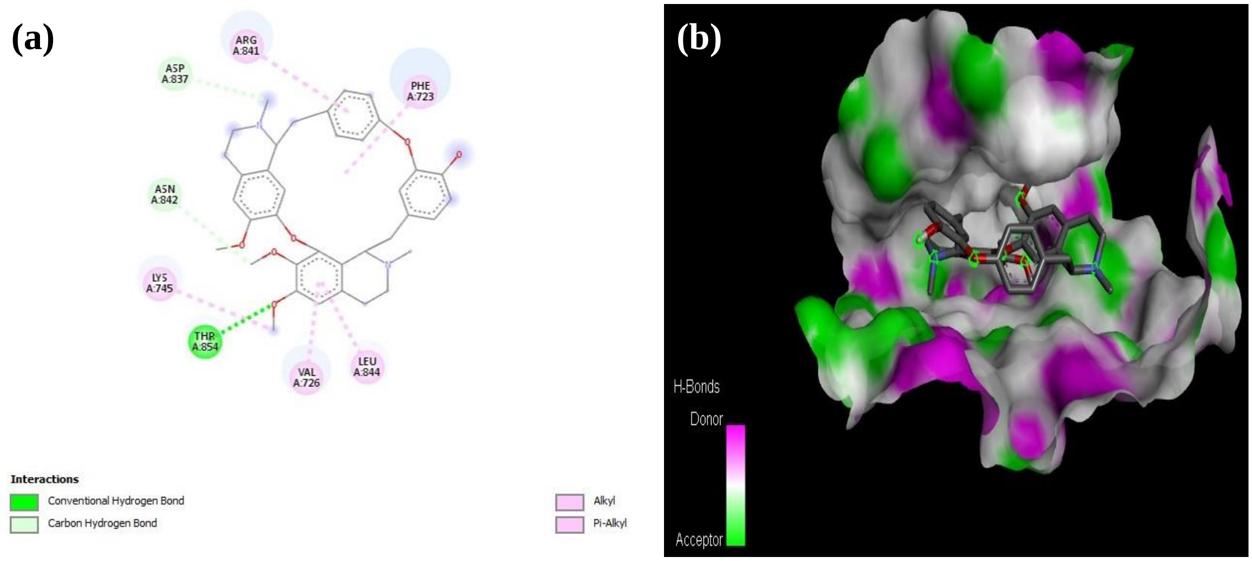

Figure S1. Interactions **(a)** and binding patterns **(b)** of berbamine with EGFR as a receptor.

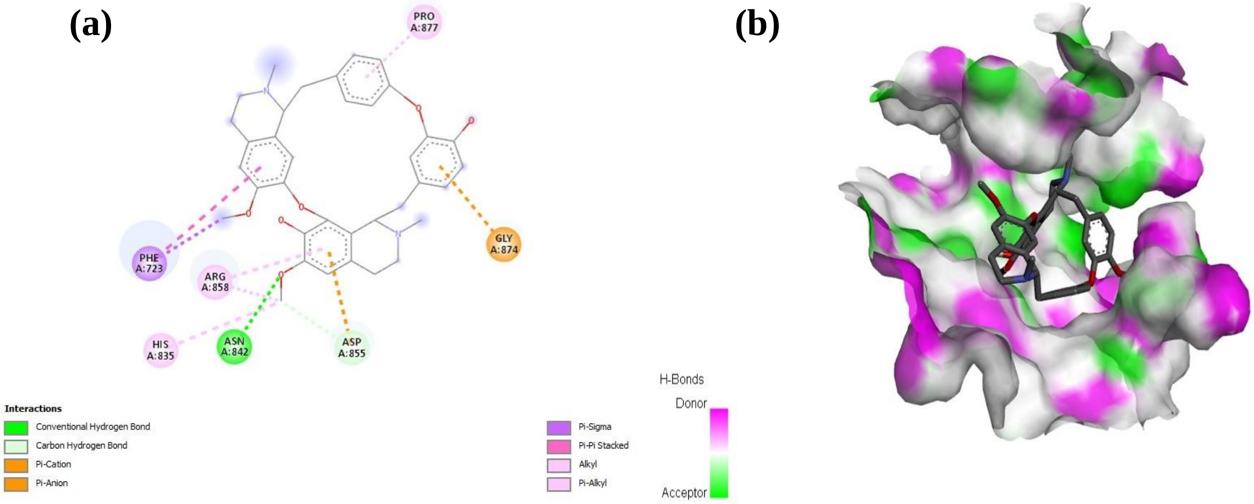

Figure S2. Interactions **(a)** and binding patterns **(b)** of obamegine with EGFR as a receptor.

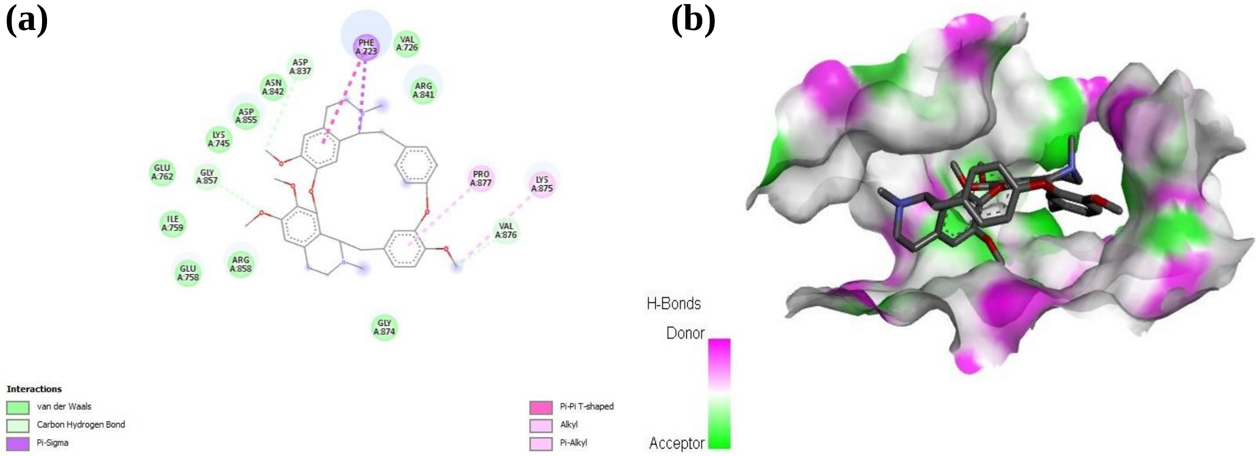

Figure S3. Interactions **(a)** and binding patterns **(b)** of isotetrandrine with EGFR as a receptor.

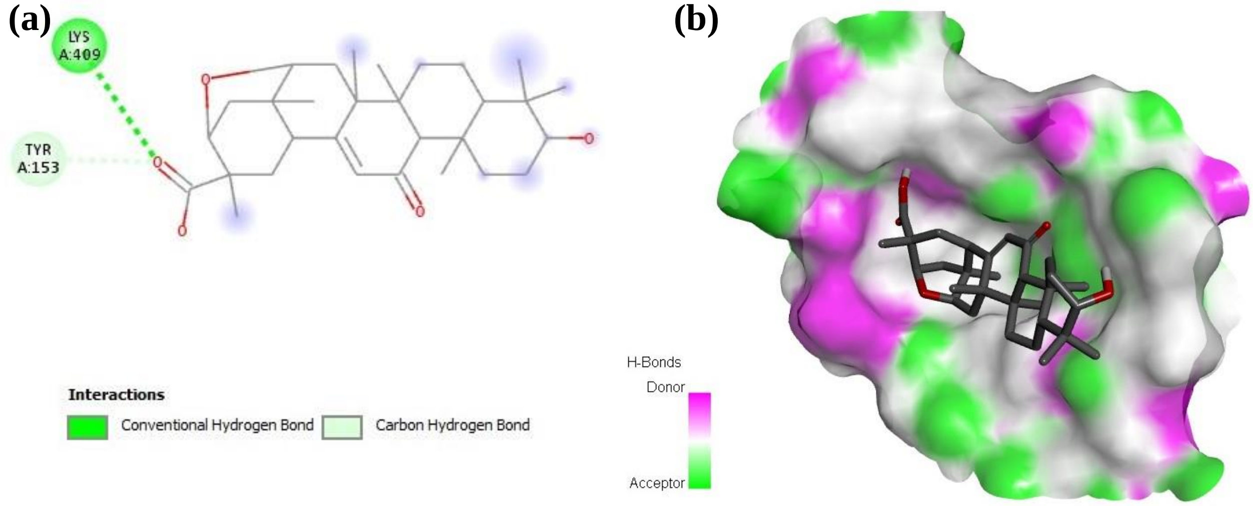

Figure S4. Interactions **(a)** and binding patterns **(b)** of liquoric acid with caspase-9 as a receptor.

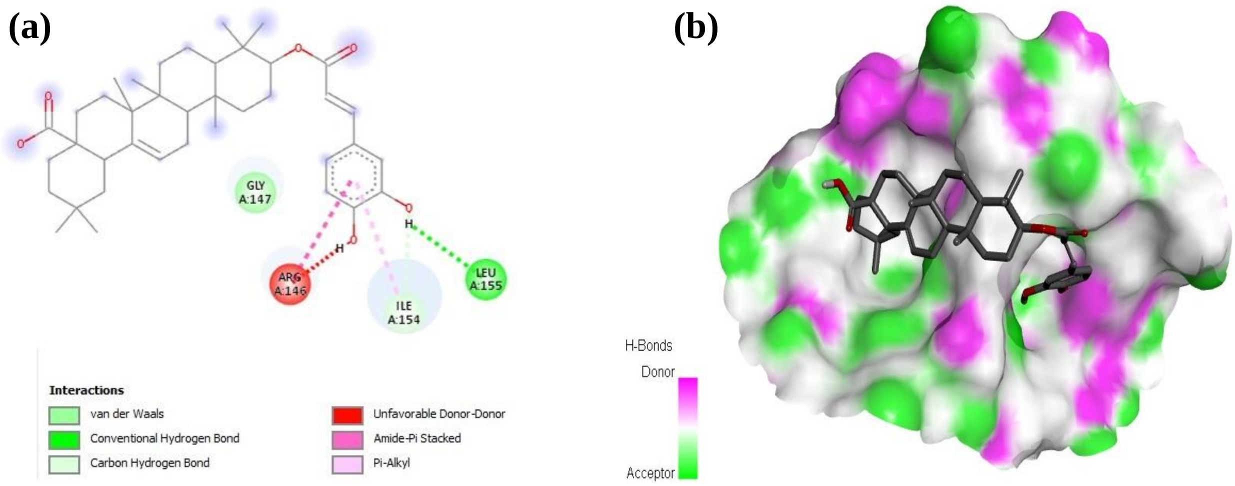

Figure S5. Interactions **(a)** and binding patterns **(b)** of 3-O-caffeoyloleanolic acid with caspase-9 as a receptor.

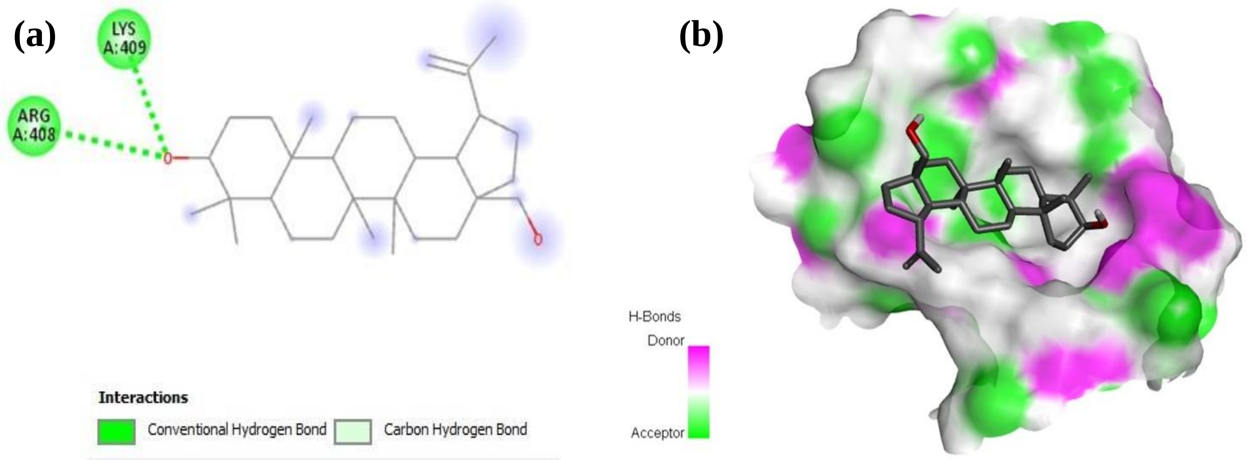

Figure S6. Interactions **(a)** and binding patterns **(b)** of betulin with caspase-9 as a receptor.

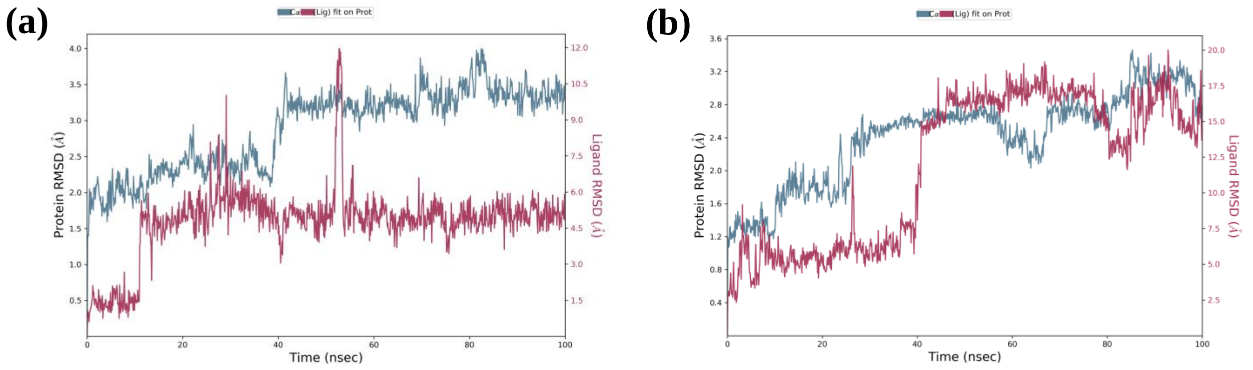

Figure S7. Root-mean-square-deviation (RMSD) values of liquoric acid and limonin with receptor proteins (replicate). (a) RMSD of the C-alpha atoms of EGFR and liquoric acid; (b)

RMSD of the C-alpha atoms of caspase-9 and limonin with time. The variation of protein RMSD is shown on left Y-axis through time. The variation of ligand RMSD is shown on right Y-axis through time.

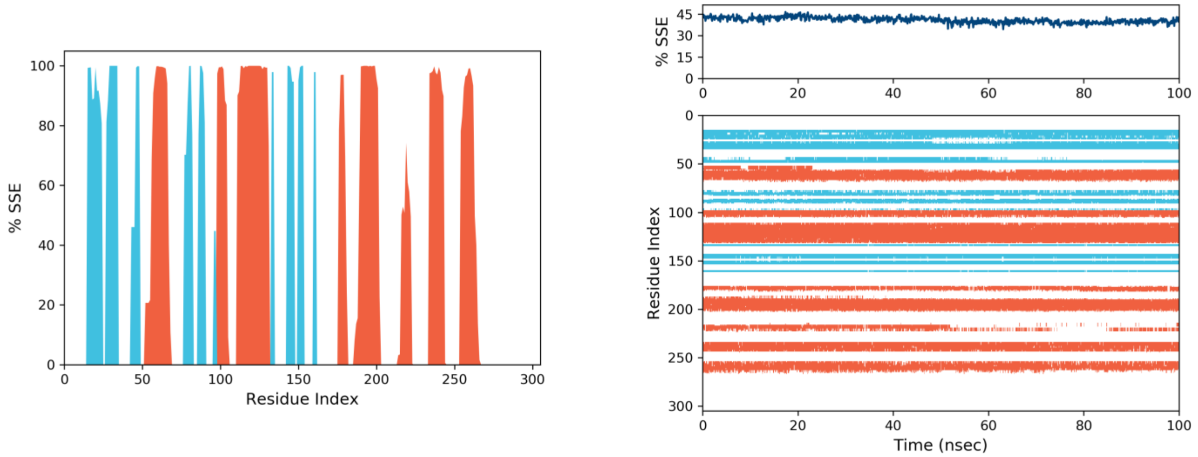

Figure S8. Protein secondary structure element (SSE) distribution by residue index throughout the protein structure of EGFR. Red indicates α-helices, and blue indicates β-strands.

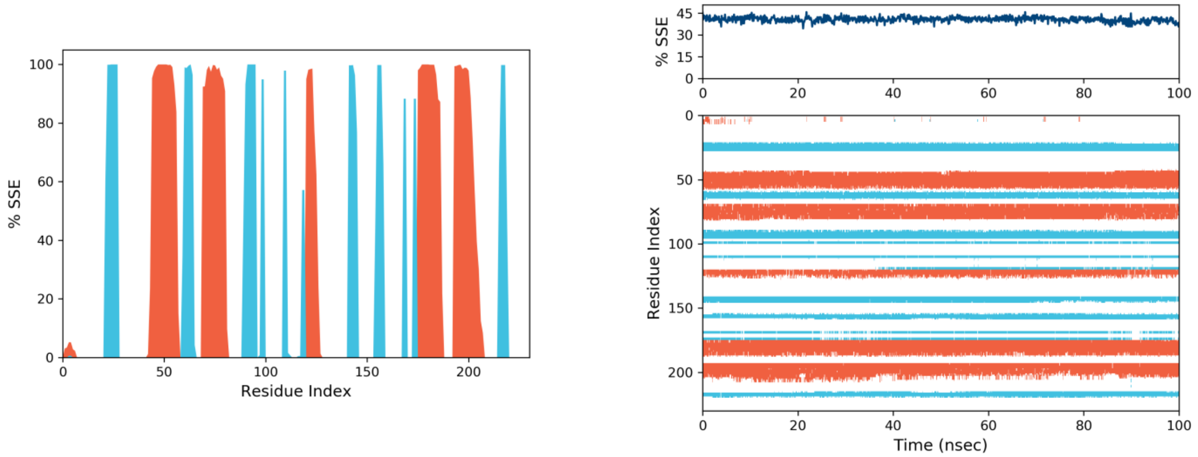

Figure S9. Protein secondary structure element (SSE) distribution by residue index throughout the protein structure of caspase-9. Red indicates α-helices, and blue indicates β-strands.

Table S1. MM-GBSA binding energy calculations of liquoric acid with EGFR after every ten nanoseconds from molecular dynamics simulation trajectories

	dG_Bind	dG_Bind_Coulomb	dG_Bind_Covalent	dG_Bind_Hbond	dG_Bind_Lipo	dG_Bind_Packing	dG_Bind_Solv_GB	dG_Bind_vdW
0 ns	-89.4532469	-20.1964304	2.161522281	-10.1137858	-20.48478976	-0.874625398	33.1668548	-69.11199264
10 ns	-71.70460897	-22.8730964	18.3044212	-11.18695775	-19.82953852	0	31.1991118	-62.3185493
20 ns	-64.73205608	-26.3937903	10.45174836	-13.01456948	-18.55079646	0	41.0627249	-72.28737309
30 ns	-77.01273104	-16.5379009	7.429698204	-8.746238974	-17.55930011	0	27.6459617	-61.24495091
40 ns	-78.41969191	-17.0373039	1.393327837	-13.98027136	-15.13217789	0	13.2052645	-54.86853111
50 ns	-63.20637707	-21.719478	4.044353271	-14.16886138	-19.19172292	0	24.2739922	-66.4446602
60 ns	-66.86487702	-19.1934702	8.898709355	-9.426649177	-17.32710599	0	50.2936171	-64.10997808
70 ns	-74.92710058	-20.1578485	19.92234824	-9.974762907	-19.04454548	0	24.758254	-60.43054597
80 ns	-72.73387237	-19.7152887	13.90699932	-8.789307757	-23.51032154	0	26.4484405	-75.0743942
90 ns	-65.5902566	-28.5457742	-0.507859506	-10.53495629	-17.23250288	0	36.7609266	-53.53009042
100 ns	-63.75442469	-18.9139355	1.702199933	-9.047595023	-13.09518802	0	44.8841973	-38.28410334
Average	-71.67265848	-21.025847	7.973406227	-10.81672326	-18.26890814	-0.0795114	32.15448595	-61.60956084
STD	8.02848325	3.691671738	7.041174456	2.023717336	2.74630482	0.26370948	10.59411367	10.18850401

Table S2. MM-GBSA binding energy calculations of limonin with caspase-9 after every ten nanoseconds from molecular dynamics simulation trajectories

	dG_Bind	dG_Bind_Coulomb	dG_Bind_Covalent	dG_Bind_Hbond	dG_Bind_Lipo	dG_Bind_Packing	dG_Bind_Solv_GB	dG_Bind_vdW
0 ns	-38.48150982	-2.045525571	1.524766066	-0.708080236	-10.96317901	-1.979992113	9.320844427	-33.63034338
10 ns	-33.27370911	1.401079388	2.91559889	-0.66148121	-12.87645416	-1.518959774	2.392908781	-24.92640102
20 ns	-37.4476948	-3.484088356	1.452245624	-0.309504943	-16.07859424	-1.646058343	7.332633294	-24.71432784
30 ns	-44.67387539	-1.387291188	1.472949032	-0.969665941	-17.50781289	-0.881713868	5.231455236	-30.63179577
40 ns	-34.01940364	-9.570164188	-0.00833352	-0.50334483	-12.70100777	-3.29E-06	12.28497313	-23.52152318
50 ns	-41.24353874	-8.442853258	0.481719277	-5.48E-05	-16.3014577	-1.435503429	14.37882339	-29.92421224
60 ns	-37.08134206	-12.68664261	1.784337561	-0.092616354	-13.26453885	-1.603812308	17.22402872	-28.44209822
70 ns	-43.80103092	-10.02575775	3.147491275	-0.269068042	-15.6514282	-2.753428008	13.30061719	-31.54945738
80 ns	-34.43718389	0.843106825	0.0732163	-0.023953834	-15.54084265	-2.177260469	5.112541262	-22.72399133
90 ns	-61.03649353	-8.085138735	0.627802541	-0.308549345	-26.27179914	-1.541356551	12.25940158	-37.71685388
100 ns	-39.38187807	-8.775203312	-0.18089529	-0.057560907	-14.43265273	-0.001529932	13.30091165	-29.23494755
Average	-40.44342363	-5.659861705	1.208263432	-0.354898222	-15.59906976	-1.412692553	10.19446715	-28.81963198
STD	7.796708821	4.850332882	1.131380724	0.319831408	4.029805484	0.842159578	4.638221519	4.602304461
